# The influence of message framing and time metaphors in green advertising on consumer effects: an examination based on the mediating role of approach-avoidance motivation

**DOI:** 10.3389/fpsyg.2025.1552963

**Published:** 2025-02-27

**Authors:** Mingfang Dong, Danyang Cao, Tianli Liu

**Affiliations:** School of Management, Xi'an University of Architecture and Technology, Xi'an, China

**Keywords:** green advertising, message framing, time metaphor, approach motivation, avoidance motivation

## Abstract

**Objectives:**

There is currently a discrepancy between consumers' understanding and practice of green consumption, resulting in inadequate levels of engagement. It is crucial for enterprises to design persuasive green advertisements to enhance consumers' willingness to make green purchases.

**Methodology:**

This research employs the perspective of time movement and the S-O-R theoretical model. Two scenario experiments were conducted alongside a questionnaire survey to examine the effects of green advertising message framing (gain vs. loss), time metaphors (ego-moving vs. time-moving), and approach-avoidance motivation on consumers' willingness to make green purchases.

**Findings:**

When green advertisements utilize a gain-framing, ego-moving metaphor effectively enhance consumers' willingness to purchase. Conversely, loss-framing paired with time-moving metaphor better promote green consumption behaviors. Approach and avoidance motivations mediate the effects between message framing and time metaphors.

**Conclusion:**

Enterprises should consider the matching effects of different information types when designing green advertisements. Specifically, aligning gain-framing with ego-moving metaphor and loss-framing with time-moving metaphor can significantly enhance consumer purchase intentions. Additionally, marketers should focus on consumers' psychological motivations, as approach-avoidance motivation affects the impact of advertising message combinations on purchasing willingness.

**Implications:**

The findings elucidate the psychological pathways influencing consumers' green purchasing decisions, assisting enterprises in optimizing advertising message strategies and offering theoretical and practical recommendations for effective green advertising design.

## 1 Introduction

In response to persistent environmental pollution, the concepts of green, low-carbon, and sustainable consumption are increasingly recognized by the public. Companies are progressively transitioning to green production methods and actively promoting environmentally friendly products to foster sustainable consumer behavior. Nonetheless, despite these initiatives, many companies report that the outcomes of their green marketing efforts are unsatisfactory. Consumer engagement with green consumption remains relatively low, and further guidance is needed to enhance the intention to purchase green products and fully realize the potential of green consumption. Consequently, it is crucial to identify effective marketing strategies that promote green consumption behavior.

Currently, sustainable consumption is receiving increasing attention from academia (Homar and Cvelbar, 2021). Previous research has highlighted that consumers' perceptions of environmental issues play a crucial role in shaping their green purchase intentions (Shen and Wang, 2022). Green information, as an external environmental stimulus, significantly influences consumers' psychological cognition, thereby affecting their decision-making processes (Wu et al., 2024). Companies often leverage green advertisements to communicate the attributes of eco-friendly products and shape consumer perceptions (Santa and Drews, 2023). These advertisements typically employ distinct message framing strategies. For instance, Liby's slogan, “Caring for Your Health, Making the World Cleaner,” conveys a benefit-focused message, emphasizing the advantages of purchasing green products for personal health and environmental protection. In contrast, Bamboo Fairy's message, “The environment is being polluted, forests are being cut down, traditional bleached tissues may harm the human body,” employs a loss-focused frame, highlighting the risks of using non-eco-friendly products. While previous studies on message framing have explored factors such as emotions (Stadlthanner et al., 2022), self-esteem (Wang et al., 2022), and mindset (Su and Li, 2024a), as well as presentation strategies like advertising appeals (Sheng et al., 2019) and scarcity (Roy and Sharma, 2015), they have largely overlooked the role of temporal cues, such as time metaphors. Time metaphors, which represent individuals' psychological perceptions of time, are categorized into ego-moving and time-moving types (Zheng et al., 2015). Previous studies have examined the influence of these metaphors on consumer behavior (Xu and Chen, 2020) and demonstrated their association with the valence of events—positive events evoke ego-moving metaphor, while negative events trigger time-moving metaphor (White et al., 2011). However, the role of time metaphors in green advertising remains underexplored, necessitating further investigation into their impact on green purchase intentions.

In summary, this research, based on the perspective of temporal movement and the S-O-R (Stimulus-Organism-Response) theoretical model, explores the matching effects of green advertisement message framing and time metaphors on the effectiveness of consumers' green purchase intentions. Specifically, the study addresses the following two questions:

How do green advertisement message framing (gain vs. loss) and time metaphors (ego-moving vs. time-moving) combine to enhance consumers' green purchase intentions?

How do approach and avoidance motivations mediate the matching effects between green advertisement message framing and time metaphors on green purchase intentions?

This study innovatively links the time cues (time metaphors) embedded in green advertising to message framing and introduces approach and avoidance motivations. The objective is to construct a mechanism that elucidates the influence of message framing and time metaphors in green advertising on consumers' green purchase intentions, thereby identifying the optimal matching combinations that enhance the effectiveness of green advertising on consumers' willingness to engage in green purchasing. Furthermore, this research delineates the psychological pathways through which different green advertising messages influence consumers' intentions to purchase green products. This not only offers a new perspective on the study of the persuasive effects of green advertising but also provides optimal design combinations and practical recommendations for companies seeking to promote green consumption behaviors and to develop effective green marketing strategies.

## 2 Literature review and research hypotheses

### 2.1 Green advertising message framing: gain-framing vs. loss-framin*g*

The concept of message framing was introduced by Tversky and Kahneman (1981), who demonstrated that the presentation of identical content can lead to varying consumer preferences and decisions depending on how it is framed (Kahneman and Tversky, 1979). Message framing is typically categorized into two types: gain-framing and loss-framing (Levin et al., 1998; Florence et al., 2022). Gain-framing emphasizes the positive outcomes or benefits associated with a purchase, whereas loss-framing highlights the negative consequences of not making the purchase.

Currently, there is no consensus among scholars regarding which framing strategy is more effective in encouraging green consumption. Some studies suggest that gain-framing is more impactful in enhancing green purchase intentions. For example, Chi et al. (2021) found that combining gain-framing with factual climate change and carbon offset information significantly increased purchase intentions for carbon offset products. Similarly, Grappi et al. (2024) reported positive outcomes of gain-framing in promoting sustainable fashion. Conversely, other researchers argue that loss-framing is more effective in driving green consumption. Amatulli et al. (2019) demonstrated that negative framing outperformed positive framing in promoting eco-friendly behaviors. This finding aligns with loss aversion theory, as Homar and Cvelbar (2021) empirically showed that loss-framing has a stronger influence on consumers' willingness to purchase green products than gain-framing.

To address these discrepancies, some studies have introduced moderating variables. Fu and Gao (2023) found that gain-framing is more effective for consumers with strong eco-friendly attitudes, while loss-framing is more persuasive for those with weaker attitudes. Zhang et al. (2023) explored how framing interacts with social contexts, revealing that loss-framing is more effective in narrative contexts, whereas gain-framing works better in performance-based settings. Furthermore, Su and Li (2024a) investigated the interplay between framing and mindsets, showing that consumers with fixed mindsets respond better to loss-framing, while those with growth mindsets prefer gain-framing.

### 2.2 Time metaphors: ego-moving vs. time-moving

Time is an abstract concept often understood through spatial metaphors. For instance, when stating, “The plane will take off in 5 min,” spatial constructs are used to represent temporal ones. Clark (1973) termed this phenomenon the “time-space metaphor,” which encompasses two types: ego-moving (where individuals perceive themselves as moving toward an event) and time-moving (where events are perceived as moving toward individuals; Duffy and Feist, 2014). For example, “We are about to start the National Day holiday” illustrates an ego-moving metaphor, while “The National Day holiday is coming up soon” reflects a time-moving metaphor.

Consumers frequently employ such time metaphors to interpret their experiences (Zhang and Ma, 2022), prompting researchers to examine their influence on consumer psychology (Chan and Maglio, 2020; Chan and Saqib, 2022). Factors such as spatial movement experience (Boroditsky and Ramscar, 2002), personality differences (Duffy and Evans, 2017), emotions (Richmond et al., 2012), and language culture (Lai and Boroditsky, 2013) can all influence metaphor choice. Margolies and Crawford (2008) found that participants imagining negative events leaned toward time-moving metaphor, while those envisioning positive events favored ego-moving metaphor. Lee and Ji (2014) demonstrated that positive future predictions typically evoke an ego-moving perspective, whereas negative predictions bring forth a time-moving perspective. Overall, positive events evoke an ego-moving perspective, while negative events align with a time-moving perspective.

With the deepening of research on time metaphors, scholars in the field of consumer behavior have discovered that time metaphors can shape information and effectively communicate it to consumers, serving as a critical factor in explaining consumer behavior. Consequently, an increasing number of researchers are focusing on the relationship between consumers and their subjective perception of time (time metaphors), believing that time metaphors influence individual decision-making and judgment processes. For instance, Xu and Chen (2020) explored the relationship between time metaphors and regulatory focus, finding that consumers in the ego-moving metaphor (as opposed to the time-moving metaphor) demonstrate a stronger preference for high-risk financial products when anticipating a positive event. Xu et al. (2024) found that ego-moving (vs. time-moving) metaphors increase consumers' perceived temporal distance from a target event, subsequently influencing their purchase decisions. Similarly, Su and Li (2024b) examined the relationship between time metaphors and tourists' willingness to act, revealing that matching time-moving (vs. ego-moving) metaphors with close-up (vs. distant) destination images elicited more favorable tourist responses. Li and Ma (2024) demonstrated that time metaphors affect the persuasiveness of informational appeals, with ego-moving (vs. time-moving) metaphors combined with rational (vs. emotional) UGC (user-generated content) enhancing consumers' visitation intentions.

Chang and Yen (2013) argued that using metaphors in advertising can produce favorable advertising effects; advertisements that include metaphors are more effective than those without, regardless of the type of metaphor. The use of time metaphors in green advertising holds substantial significance. Ego-moving metaphors can evoke positive consumer visions of a sustainable future by suggesting the impending availability of green products, thereby conveying a sense of foresight and a call to action. Consumers are likely to believe that engaging in green consumption can contribute to a better future, thereby increasing their purchase intentions. In contrast, time-moving metaphors can heighten consumers' awareness of impending environmental crises by emphasizing that “the green choice is urgent,” thus reinforcing the necessity of selecting green products. Consequently, this study proposes that time metaphors embedded within green advertisements, as vehicles for information transmission, similarly influence consumers' processing of information related to green advertisements and green products, thereby impacting their green purchase intentions.

### 2.3 The matching mechanism between message framing and temporal metaphors

Consumers' information processing ability can enhance the persuasive effect of advertisements, thereby increasing their green purchase intentions (Luo et al., 2023). Reber et al. (2004) found that the consistency effect generated by matching factors that influence consumers' information processing ability can promote the fluency of information processing. This improves the speed and quality of consumers' information evaluation, thereby enhancing their cognitive appraisal of brands and advertisements, which subsequently increases their purchase intentions. As critical components of green advertising information delivery, message framing and time metaphors not only directly influence the interpretation of information but also indirectly regulate consumers' decision-making behavior by affecting their psychological perceptions and emotional responses.

Zheng et al. (2015) indicated that the emotional valence associated with an event relates to the temporal perspective through which individuals interpret it. Positive events or emotions are typically associated with ego-moving metaphor, while negative ones are connected to time-moving metaphor (Lee and Ji, 2014). When individuals are faced with positive information, they tend to perceive themselves as moving closer to a favorable outcome, such as “heading toward the light,” thereby enhancing their expectation of positive future results. In contrast, time metaphors associated with negative events or emotions imply that, when faced with negative information, individuals often feel that bad consequences are gradually approaching them, such as “a great disaster is looming,” which triggers a psychological tendency to avoid potential threats. Prospect Theory suggests that gain-framing lead consumers to perceive that purchasing green products or services will yield “certain gains,” which may include health benefits, savings, and environmental advantages. These “gains” carry a positive connotation, encouraging consumers to focus on favorable messages presented through gain-framing. In contrast, loss-framing lead consumers to believe that failing to choose green products or services could result in “potential losses,” including health risks, higher costs, and environmental harm. These “losses” convey negative implications, causing consumers to concentrate on unfavorable messages under loss-framing. Therefore, there is a high degree of consistency between the psychological focus guided by the message framing and the time metaphor.

Therefore, this study proposes that when the message framing of green advertisements is matched with different time metaphors, the consistency effect of information matching can enhance consumers' cognitive fluency and depth of understanding regarding green advertisements and green products. This, in turn, improves consumers' information-processing ability and strengthens their green purchase intentions. Specifically, when green advertisements convey positive, benefit-oriented information, matching with ego-moving metaphor leads consumers to perceive that engaging in green consumption behaviors will bring them and the environment closer to positive outcomes. Conversely, when green advertisements convey negative, loss-oriented information, matching with time-moving metaphor causes consumers to believe that green consumption behaviors can help prevent negative outcomes for themselves and the environment. Thus, the following hypotheses are proposed:

H1: There is a matching effect between the message framing and time metaphors.
H1a: When green advertisements adopt a gain-framing, ego-moving metaphor (vs. time-moving metaphor) are more effective in enhancing green purchase intentions.
H1b: When green advertisements adopt a loss-framing, time-moving metaphor (vs. ego-moving metaphor) are more effective in enhancing green purchase intentions.

### 2.4 The mediating role of approach motivation and avoidance motivation

Motivation is an internal force that drives and sustains individual activities (Elliot, 2008). It reflects the interaction between individuals and their environment, functioning as a core mechanism for seeking rewards and avoiding harm to adapt to external conditions. Motivation comprises two primary dimensions: approach motivation, which is triggered by positive stimuli and encourages the pursuit of rewards, and avoidance motivation, which arises from negative stimuli and fosters the avoidance of potential losses (Carver and White, 1994). According to Elliot (1999), the activation of approach and avoidance motivations is influenced by both the situational stimuli and their valence (positive or negative). Positive perceptions activate approach motivation, while negative perceptions evoke avoidance motivation (Michaelsen and Esch, 2023). When individuals perceive that a certain event or behavior will lead to positive outcomes, they are more likely to develop approach motivation. Conversely, if individuals perceive that an event or behavior will result in negative consequences, they are inclined to develop avoidance motivation to mitigate potential losses or harm.

Consumers' psychological motivation is a critical variable in explaining their decision-making behavior and plays an essential role in analyzing and predicting their choices. Notably, motivation matching has been shown to enhance the persuasiveness of information. Specifically, compared to information that conflicts with an individual's motivation, information that aligns with situational factors consistent with an individual's motivation (rather than individual differences) yields a stronger motivation-matching effect. As previously discussed, gain-framing communicate positive valence information, emphasizing the benefits of “green consumption,” which in turn activates consumers' approach motivation and promotes their engagement in green behaviors. In contrast, loss-framing highlight the negative consequences of not engaging in green consumption, thereby activating avoidance motivation and deterring behaviors inconsistent with sustainable practices. Consequently, different message framings stimulate corresponding approach or avoidance motivations, guiding consumers' adoption of green consumption behaviors. Similarly, time metaphors are closely associated with individuals' approach-avoidance motivations. Positive event valence is typically linked to the ego-moving perspective, while negative event valence is associated with the time-moving perspective, aligning with the positive or negative stimuli that activate approach or avoidance motivations, respectively. Research indicates that anticipated positive events are more likely to activate ego-moving metaphor aligned with promotion focus, whereas anticipated negative events tend to evoke time-moving metaphor associated with prevention focus (Xu and Chen, 2020). Based on the theory of regulatory focus, promoting focus toward positive outcomes and facilitating approach behavior, preventing focus emphasizing negative outcomes and promoting avoidance behavior, illustrating the varying motivations that arise across different contexts (Higgins, 1997). Accordingly, this study suggests that ego-moving metaphor paired with gain-framing activate approach motivation, while time-moving metaphor combined with loss-framing activate avoidance motivation.

The S-O-R (Stimulus-Organism-Response) theory has been widely applied to consumer decision-making research (Zhao et al., 2024). This framework posits that external stimuli (S) influence an individual's internal psychological state (O), which in turn affects behavioral responses (R). In the context of green advertisements, stimuli such as gain-framing paired with ego-moving metaphor communicate positive messages, thereby enhancing approach motivation and promoting “green consumption.” Conversely, loss-framing combined with time-moving metaphor convey negative messages, activating avoidance motivation and discouraging non-participation in green consumption. Based on these insights, the study proposes the following hypotheses:

H2: Approach-avoidance motivation mediates the matching effect between message framing and time metaphors.
H2a: Approach motivation mediates the effect of gain-framing and ego-moving metaphor on consumers' green purchase intentions.
H2b: Avoidance motivation mediates the effect of loss-framing and time-moving metaphor on consumers' green purchase intentions.

**Figure 1:**
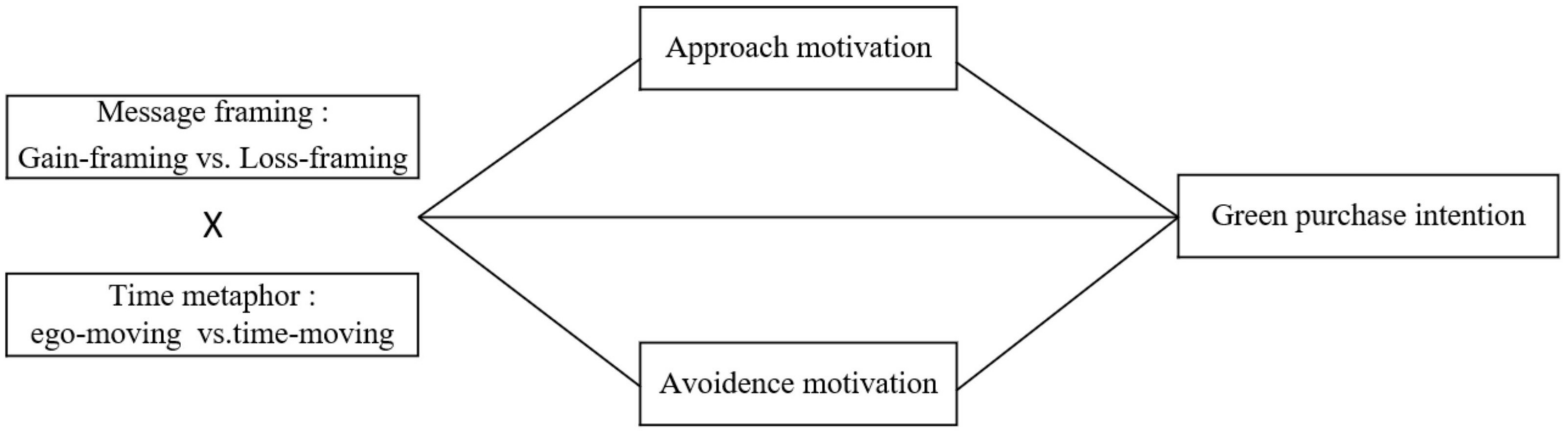
Theoretical model.

Based on the above literature review and theoretical hypotheses, the research framing of this study is illustrated in Figure 1.

## 3 Methodology

### 3.1 Data collection method

This study investigates how message framing and time metaphors influence consumers' willingness to engage in green purchasing, with a focus on the mediating roles of approach and avoidance motivations. A pre-experiment and two formal experiments were designed for this research, using random sampling methods to collect experimental data through the “Credamo” online research platform. The “Credamo” platform features a rich sample pool that encompasses a diverse range of demographics, allowing for precise targeting and ensuring that the data source is authentic and reliable. In the pre-experiment, a total of 60 participants were recruited, while Experiments 1 and 2 involved the recruitment of 200 participants each.

### 3.2 Measurement

This study measured message framing, time metaphors, approach and avoidance motivations, as well as consumers' willingness to engage in green purchasing through a comprehensive questionnaire. To ensure validity and reliability, all questionnaire items were derived from established, validated scales, as detailed in Table 1.

**Table 1:** Measurement scales.

**Sr. No**	**Variable**	**Sub-variable**	**No. of items**	**Reference of the study**
1	Message framing		06	(Meyers-Levy and Maheswaran, 2004) (Sheng et al., 2019)
2	Time metaphor		04	(Núñez et al., 2006) (Liu et al., 2023)
3	Motivation	Approach motivation	09	(Carver and White, 1994)
		Avoidance motivation	06	
4	Green purchasing intention		04	(Laukov, 2013)

### 3.3 Data analyses techniques

This study utilized SPSS 26.0 and AMOS 26.0 software for data analysis to explore the potential relationships among the variables. Firstly, we conducted manipulation checks on message framing and time metaphors, validating the success of the manipulations using *t*-tests. Secondly, we performed tests for main effects and simple effect analyses to confirm the matching effects of message framing and time metaphors on consumers' willingness to engage in green purchasing. Finally, we conducted mediation effect analyses to clarify the mediating roles of approach and avoidance motivations. This research analyzed the overall model paths, elucidating the impact of the matching combinations of message framing and time metaphors in green advertisements on consumers' willingness to engage in green purchasing.

### 3.4 Pre-experiment

To determine the experimental materials and stimuli, this study used focus group interviews to identify 10 common green products: new energy vehicles, solar water heaters, eco-friendly bamboo paper, fluorine-free refrigerators, mercury-free alkaline batteries, formaldehyde-resistant latex paint, LED bulbs, phosphate-free laundry detergent, organic whole milk, and natural cosmetics. Two surveys were then released on the “Credamo” online survey platform to test consumers' familiarity with these products, each involving 30 participants. The first survey asked participants to classify the ten products as green. The results showed recognition rates for new energy vehicles, solar water heaters, eco-friendly bamboo paper, fluorine-free refrigerators, mercury-free alkaline zinc-manganese batteries, formaldehyde-resistant odorless latex paint, LED bulbs, phosphate-free laundry detergent, organic whole milk, and natural cosmetics to be 96.7, 93.3, 93.3, 80, 76.7, 66.7, 70, 83.3, 63.3, and 56.7%, respectively. Since the recognition rates for formaldehyde-resistant odorless latex paint, organic whole milk, and natural cosmetics were relatively low, these three green products were excluded from the second survey questionnaire.

The second survey aimed to assess participants' familiarity with seven green products: new energy vehicles, solar water heaters, eco-friendly bamboo paper, fluorine-free refrigerators, mercury-free alkaline zinc-manganese batteries, LED bulbs, and phosphate-free laundry detergent. Familiarity was rated from 1 (very unfamiliar) to 7 (very familiar), with higher familiarity indicating better understanding of these products and reducing comprehension biases in the main experiment. The results showed the following levels of familiarity with the seven green products: M new energy vehicles = 5.933, M solar water heaters = 5.800, M eco-friendly bamboo paper = 5.933, M fluorine-free refrigerators = 5.033, M mercury-free alkaline zinc-manganese batteries = 4.533, M LED bulbs = 5.367, M phosphate-free laundry detergent = 5.767. Among these, participants were relatively more familiar with new energy vehicles and eco-friendly bamboo paper. Conversely, the familiarity with mercury-free alkaline zinc-manganese batteries was relatively low.

Combining the data from the two survey questionnaires and considering the diversity of green products in real life, this study selected new energy vehicles and eco-friendly bamboo paper as the stimuli for the formal experiment.

### 3.5 Experiment 1

#### 3.5.1 Experimental design

Permission must be obtained for use of copyrighted material from other sources (including the web). Please note that it is compulsory to follow figure instructions. Experiment 1 employs a 2 (message framing: gain-framing vs. loss-framing) × 2 (temporal metaphor: ego-moving vs. time-moving) between-subjects factorial design. A total of 200 participants were recruited for this experiment through the “Credamo” online survey platform. Out of the initial 200 samples: 4 samples failed the attention check item, 4 samples exhibited abnormal response times, 6 samples showed uniform responses across all items. After excluding these three categories of invalid samples, a total of 186 valid responses were obtained, resulting in an effective rate of 92%. The demographic breakdown of the valid sample is as follows: 82 male participants (44.09%) and 104 female participants (55.91%). Those aged under 18 accounted for 4 individuals (2.15%), those aged 18–25 accounted for 54 individuals (29.03%), those aged 26–35 accounted for 98 individuals (52.69%), those aged 36–45 accounted for 17 individuals (9.14%), those aged 46–55 accounted for 8 individuals (4.30%), and those aged over 55 accounted for 5 individuals (2.69%).

Experiment 1 used advertisements for eco-friendly bamboo paper sourced from WeChat, Weibo, and other media platforms. Four sets of stimuli were created to match experimental conditions, ensuring consistency in message framing, metaphors, product images, and layout while omitting product names and logos. Previous research indicates that clarifying the impacts of green products vs. traditional products on individual consumers and the natural environment allows for a distinction between gain and loss framing (Sheng et al., 2019). The content of the stimuli materials is shown in the following Table 2. Specifically, the stimulus materials for green advertising under the gain frame with an ego-moving metaphor include: “Choose eco-friendly bamboo paper. Natural bamboo fibers, antibacterial and unbleached. Adopting bamboo instead of wood, you will gradually approach a beautiful future with green mountains and clear waters.” In contrast, the stimulus materials for the gain frame with a time-moving metaphor state: “Choose eco-friendly bamboo paper. Natural bamboo fibers, antibacterial and unbleached. Adopting bamboo instead of wood brings a green, sustainable future closer.” For the loss frame with an ego-moving metaphor, the stimulus materials present: “Every time you use a traditional paper towel, you take a step closer to a 'desert planet'! Traditional paper towels contain harmful fluorescent agents and bleaches, harming your health and the environment.” Similarly, the loss frame with a time-moving metaphor states: “Every time you use a traditional paper towel, the 'desert planet' comes closer! Traditional paper towels contain harmful fluorescent agents and bleaches, posing risks to your health and damaging the ecological environment.”

**Table 2:** Introduction to the content of the material.

**Experimental materials**	**Material content**
Gain-framing, ego-moving metaphor	“Choose eco-friendly bamboo paper. Natural bamboo fibers, antibacterial and unbleached. Adopting bamboo instead of wood, you will gradually approach a beautiful future with green mountains and clear waters.”
Gain-framing, time-moving metaphor	“Choose eco-friendly bamboo paper. Natural bamboo fibers, antibacterial and unbleached. Adopting bamboo instead of wood brings a green, sustainable future closer.”
Loss-framing, ego-moving metaphor	“Every time you use a traditional paper towel, you take a step closer to a 'desert planet'! Traditional paper towels contain harmful fluorescent agents and bleaches, harming your health and the environment.”
Loss-framing, time-moving metaphor	“Every time you use a traditional paper towel, the 'desert planet' comes closer! Traditional paper towels contain harmful fluorescent agents and bleaches, posing risks to your health and damaging the ecological environment.”

#### 3.5.2 Experimental procedure

Participants were randomly assigned to one of four groups: gain-framing-ego-moving metaphor, gain-framing-time-moving metaphor, loss-framing-ego-moving metaphor, and loss-framing-time-moving metaphor. They received the following introduction: “Imagine you are planning to purchase household tissues and find an advertisement for eco-friendly bamboo pulp tissues on a new media platform.” After this, participants read the stimulus material for their group and answered questions about message framing, time metaphors, and their purchase intentions for the eco-friendly tissues.

The manipulation check for message framing in Experiment 1 referenced studies by Meyers-Levy and Maheswaran (2004) as well as Sheng et al. (2019), including six items such as “Do you think this advertisement emphasizes the benefits of using eco-friendly bamboo pulp tissues for yourself/the environment?” (1 = strongly disagree, 7 = strongly agree). The manipulation check for time metaphors referenced Núñez et al. (2006) and Liu et al. (2023), including four items such as “Do you think the scenario described in the stimulus is approaching you?” (1 = strongly disagree, 7 = strongly agree).

The measurement of green purchase intention followed Laukov's (2013) scale, which included four items such as “I am willing to collect and learn more messages about eco-friendly bamboo pulp tissues” (1 = strongly disagree, 7 = strongly agree). Finally, participants provided demographic information such as gender, age, education level, and monthly income.

#### 3.5.3 Experimental results

##### 3.5.3.1 Manipulation check

An independent samples *t*-test was performed to verify the effectiveness of the message framing manipulation. Average scores for the manipulation check items were computed to form indices for both the gain and loss-framing. The *t*-test results revealed that participants in the gain-framing group viewed the stimulus material as more oriented toward individual and environmental benefits compared to the loss-framing group (M gain-framing = 5.897, SD = 0.702; M loss-framing = 3.274, SD = 1.600; *t* = 14.584, *p* < 0.01). Likewise, participants in the loss-framing group perceived the material as being more focused on the potential losses for individuals and the environment compared to those in the gain-framing group (M gain-framing = 3.202, SD = 1.650; M loss-framing = 5.653, SD = 1.086; *t* = −11.908, *p* < 0.01). Thus, the manipulation of message framing was deemed successful. Next, a manipulation check for the time metaphors was conducted. The findings indicated that in the ego-moving metaphor condition, participants rated their perception of the ego-moving metaphor significantly higher than the time-moving metaphor (M ego-moving = 5.907, SD = 0.906; M time-moving = 4.102, SD = 1.501; *t* = 9.937, *p* < 0.01). Conversely, in the time-moving metaphor condition, participants rated their perception of the time-moving metaphor higher than that of the ego-moving metaphor (M ego-moving = 3.522, SD = 1.632; M time-moving = 5.968, SD = 1.057; *t* = −12.132, *p* < 0.01). Therefore, the manipulation of the time metaphors was successful as well.

##### 3.5.3.2 Main effect test

**Figure 2:**
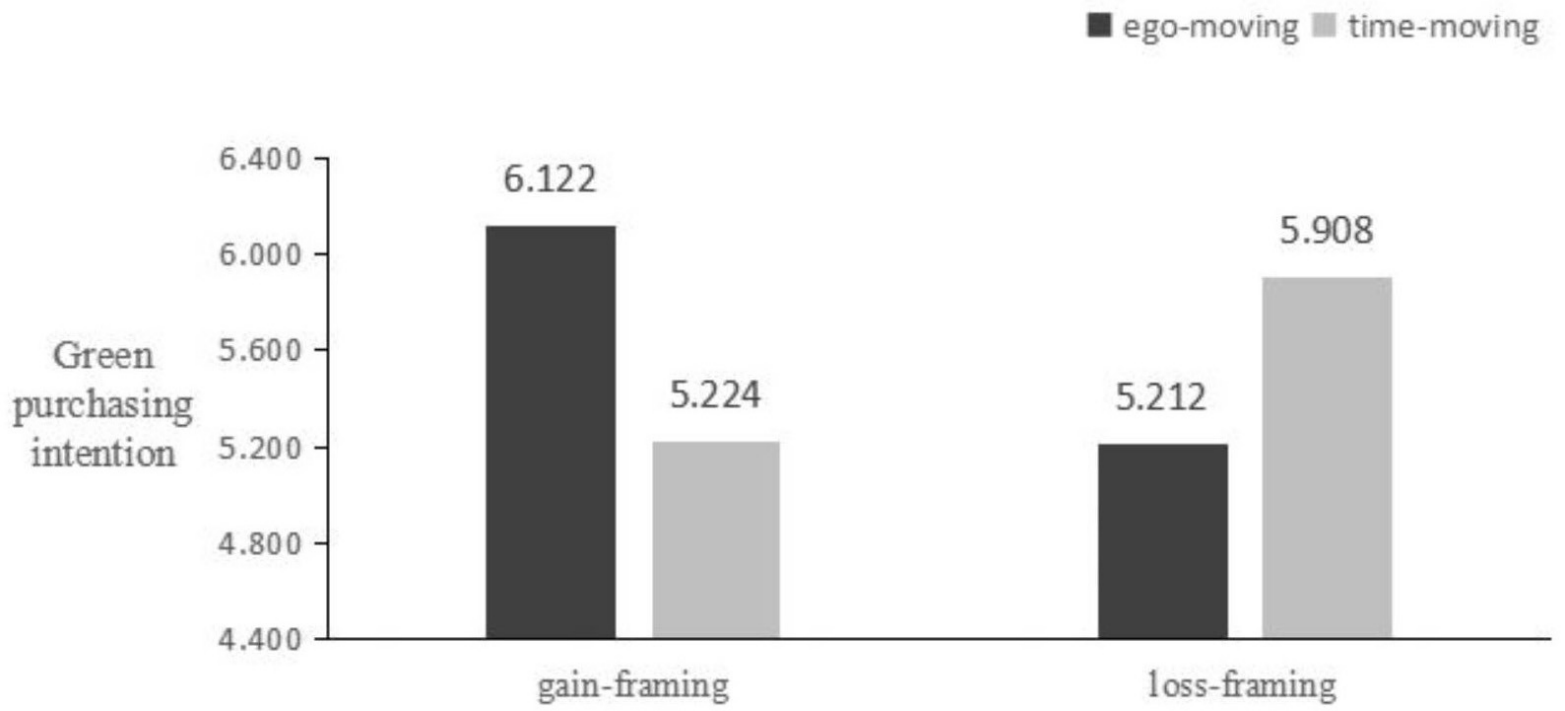
Main effect test of Experiment 1.

A two-way ANOVA was performed with message framing and time metaphors as independent variables and green purchase intentions as the dependent variable. The analysis showed that the main effect of green advertising message framing was not significant [M gain-framing = 5.692, SD = 0.883; M loss-framing = 5.571, SD = 0.903; F_(1, 185)_ = 0.856, *p* > 0.05]. Likewise, the main effect of time metaphors was also non-significant [M ego-moving = 5.667, SD = 0.843; M time-moving = 5.594, SD = 0.983; F_(1, 185)_ = 0.306, *p* > 0.05]. However, the interaction between message framing and time metaphors was significant [F_(1, 185)_ = 43.679, *p* < 0.01], as depicted in Figure 2. This suggests that the interplay between message framing and time metaphors has a significant impact on consumers' green purchase intentions, despite the main effects of each factor being non-significant when considered individually.

##### 3.5.3.3 Simple effect analysis

The experimental results showed that when green advertisements used a gain-framing, the ego-moving metaphor was more effective in increasing participants' green purchase intentions compared to the time-moving metaphor [M ego-moving = 6.112, SD = 0.551; M time-moving = 5.244, SD = 0.953; F_(1, 90)_ = 26.316, *p* < 0.01]. Conversely, when green advertisements used a loss-framing, the time-moving metaphor was more effective in enhancing participants' green purchase intentions compared to the ego-moving metaphor [M ego-moving = 5.212, SD = 0.851; M time-moving = 5.908, SD = 0.824; F_(1, 94)_ = 17.701, *p* < 0.01]. Thus, hypotheses H1, H1a, and H1b were validated. This confirms that the matching of message framing and time metaphors plays a critical role in influencing consumers' green purchase intentions.

### 3.6 Experiment 2

#### 3.6.1 Experimental design

Experiment 2 used a design like Experiment 1, with a 2 (message framing: gain-framing vs. loss-framing) × 2 (temporal metaphor: ego-moving vs. time-moving) between-subjects factorial design, and included measurements of approach and avoidance motivation. Conducted online via “Credamo,” where 200 questionnaires were distributed. After excluding those with short response times, failed attention checks, or invalid responses, a total of 180 valid samples remained (83 male, 97 female).

Unlike Experiment 1, which focused on eco-friendly products, Experiment 2 utilized new energy vehicles (NEVs) as the stimulus, maintaining consistent design principles. The stimuli materials are shown in Table 3. Specifically, the stimulus materials for green advertising under the gain-framing with an ego-moving metaphor include: “Driving a new energy vehicle is economical, green, and environmentally friendly, can effectively reduce carbon dioxide emissions and help reduce unnecessary expenses for you. Choosing new energy vehicles will gradually lead you toward a beautiful tomorrow of green and low-carbon.” In contrast, the stimulus materials for the gain-framing with a time-moving metaphor state: “Driving a new energy vehicle is economical, green, and environmentally friendly, can effectively reduce carbon dioxide emissions and reduce unnecessary expenses for you. Choosing new energy vehicles, a green and low-carbon bright future will gradually approach you.” For the loss-framing with an ego-moving metaphor, the stimulus materials present: “Driving traditional gasoline cars is not only expensive and pollutes the environment, but also harmful to health. Choosing traditional fuel vehicles will gradually lead you toward an era full of waste.” Similarly, the loss-framing with a time-moving metaphor states: “Driving traditional gasoline cars is not only expensive and pollutes the environment, and is also harmful to health. If this continues, exhaust fumes will soon overwhelm your life.”

**Table 3:** Introduction to the content of the material.

**Experimental materials**	**Material content**
Gain-framing, ego-moving metaphor	“Driving a new energy vehicle is economical, green, and environmentally friendly, can effectively reduce carbon dioxide emissions and help reduce unnecessary expenses for you. Choosing new energy vehicles will gradually lead you toward a beautiful tomorrow of green and low-carbon.”
Gain-framing, time-moving metaphor	“Driving a new energy vehicle is economical, green, and environmentally friendly, can effectively reduce carbon dioxide emissions and reduce unnecessary expenses for you. Choosing new energy vehicles, a green and low-carbon bright future will gradually approach you.”
Loss-framing, ego-moving metaphor	“Driving traditional gasoline cars is not only expensive and pollutes the environment, but also harmful to health. Choosing traditional fuel vehicles will gradually lead you toward an era full of waste.”
Loss-framing, time-moving metaphor	“Driving traditional gasoline cars is not only expensive and pollutes the environment, and is also harmful to health. If this continues, exhaust fumes will soon overwhelm your life.”

#### 3.6.2 Experimental procedure

Participants were randomly assigned to one of four experimental groups and asked to imagine a purchase scenario: “Imagine you are planning to buy a car and you find an advertisement for a new energy vehicle on a new media platform.” After this introduction, they read the relevant materials and completed manipulation checks for message framing and time metaphors, as well as measures for green purchase intentions and approach-avoidance motivation.

The manipulation checks and green purchase intention scale were the same as in Experiment 1. Approach-avoidance motivation was measured using Carver and White's (1994) Behavioral Activation and Inhibition System Scale (BAS and BIS). The avoidance motivation scale comprised seven items, while the approach motivation included nine items from the reward responsiveness and drive components. All items were rated on a seven-point Likert scale (1 = strongly disagree, 7 = strongly agree).

Finally, participants provided demographic information like gender, age, education level, and income, and received a 2-yuan reward for completing the experiment.

#### 3.6.3 Experimental results

##### 3.6.3.1 Reliability and validity analysis

This study assessed the reliability of the questionnaire scales using Cronbach's a coefficient, with the specific results presented in Table 4. The findings indicate that the Cronbach's a coefficients for approach motivation, avoidance motivation, and green purchasing intention all exceed 0.7, and are higher than the coefficients after item deletion. Additionally, the corrected item-total correlations are >0.4, suggesting that the measurement items of the scale demonstrate high stability and validity. This implies that the questionnaire accurately reflects the corresponding variables and that there is a strong correlation and stability among the measured items. This study employed the KMO test, Bartlett's sphericity test, and factor analysis to assess the suitability of the questionnaire data for information extraction. The results revealed that the standard factor loadings for the latent variables of approach motivation, avoidance motivation, and green purchasing intention were all >0.6, with a KMO value of 0.6 and a *p*-value from Bartlett's test <0.05, indicating excellent validity of the questionnaire data. Subsequently, the study further evaluated the convergent and discriminant validity of the questionnaire scales. The analysis results showed that the composite reliability (CR) for approach motivation, avoidance motivation, and green purchasing intention were 0.917, 0.926, and 0.911, respectively, all exceeding the 0.7 threshold. The average variance extracted (AVE) values were 0.551, 0.675, and 0.719, respectively, all >0.5, the specific results are shown in Table 5. Therefore, the scale demonstrates good convergent and discriminant validity, confirming the scientific rigor and reliability of the questionnaire design.

**Table 4:** Reliability scales.

**Variable**	**Variable indicators**	**Corrected item-total correlation**	**Cronbach's alpha if item deleted**	**Cronbach's α**
Approach motivation	BAS1	0.691	0.884	0.898
	BAS2	0.717	0.882	
	BAS3	0.602	0.891	
	BAS4	0.707	0.883	
	BAS5	0.691	0.884	
	BAS6	0.605	0.891	
	BAS7	0.639	0.888	
	BAS8	0.703	0.883	
	BAS9	0.603	0.891	
Avoidance motivation	BIS1	0.795	0.876	0.902
	BIS2	0.719	0.887	
	BIS3	0.713	0.888	
	BIS4	0.750	0.883	
	BIS5	0.670	0.895	
	BIS6	0.761	0.881	
Green purchasing intention	PI1	0.713	0.833	0.867
	PI3	0.734	0.825	
	PI3	0.727	0.828	
	PI4	0.706	0.836	

**Table 5:** Validity scales.

**Variable**	**Variable indicators**	**Standardized factor loading**	**CR**	**AVE**	**KMO**	**Bartlett's test of sphericity**
Approach motivation	BAS1	0.767	0.917	0.551	0.917	0.000^***^
	BAS2	0.790				
	BAS3	0.687				
	BAS4	0.783				
	BAS5	0.770				
	BAS6	0.689				
	BAS7	0.721				
	BAS8	0.779				
	BAS9	0.686				
Avoidance motivation	BIS1	0.869	0.926	0.675	0.892	0.000^***^
	BIS2	0.812				
	BIS3	0.807				
	BIS4	0.831				
	BIS5	0.767				
	BIS6	0.841				
Green purchasing intention	PI1	0.844	0.911	0.719	0.793	0.000^***^
	PI2	0.854				
	PI3	0.849				
	PI4	0.839				

^*^p < 0.05, ^**^p < 0.01, ^***^p < 0.001, ns represents *p* > 0.05.

##### 3.6.3.2 Manipulation check

An independent samples *t*-test was performed to assess the effectiveness of the message framing manipulation. The results indicated that participants in the gain-framing group viewed the stimulus material as more centered on benefits for themselves and the environment compared to those in the loss-framing group (M gain-framing = 5.900, SD = 0.644; M loss-framing = 3.247, SD = 1.511; *t* = 15.282, *p* < 0.01). Additionally, participants in the loss-framing group perceived the material as more focused on the potential losses for themselves and the environment compared to the gain-framing group (M gain-framing = 3.208, SD = 1.530; M loss-framing = 5.678, SD = 0.848; *t* = −13.549, *p* < 0.01). This confirms that the manipulation of message framing was effective. Next, a manipulation check for the time metaphors used in the green advertising was conducted. The findings revealed that in the ego-moving metaphor condition, participants rated the ego-moving metaphor significantly higher than the time-moving metaphor (M ego-moving = 5.950, SD = 0.923; M time-moving = 3.645, SD = 1.623; *t* = 11.837, *p* < 0.01). Conversely, in the time-moving metaphor condition, participants rated the time-moving metaphor significantly higher than the ego-moving metaphor (M ego-moving = 3.758, SD = 1.485; M time-moving = 5.925, SD = 0.944; *t* = −11.687, *p* < 0.01). Thus, the manipulation of the time metaphors was also effective.

##### 3.6.3.3 Main effect test

**Figure 3:**
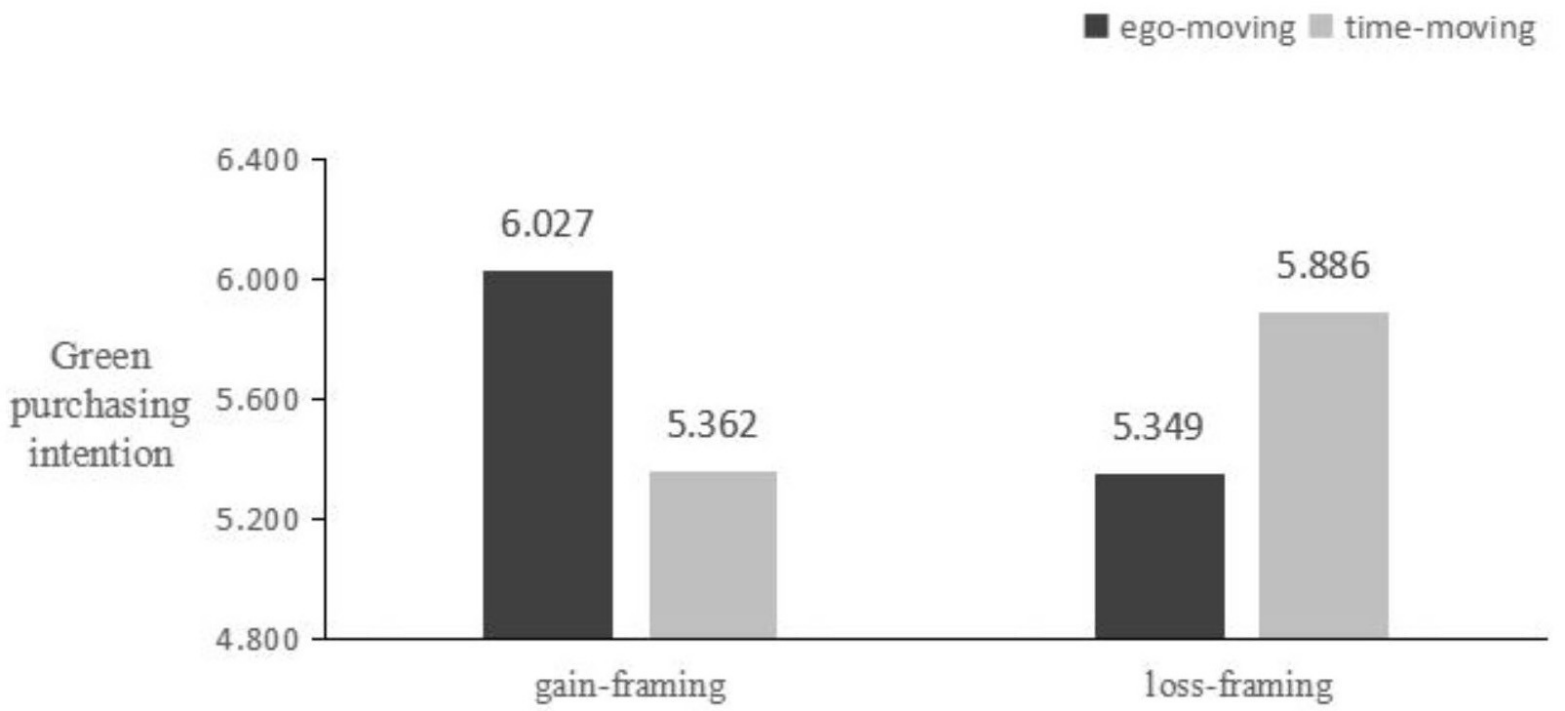
Main effect test of Experiment 2.

The reliability of the green purchase intention scale was found to be a = 0.867. A general linear model (GLM) was employed to examine the matching effect between message framing and time metaphors. The results indicated that this matching effect was significant [F_(1, 179)_ = 32.912, *p* < 0.01], as illustrated in Figure 3.

##### 3.6.3.4 Simple effect analysis

The results showed that when green advertisements used the gain-framing the ego-moving metaphor was more effective in increasing participants' green purchase intentions compared to the time-moving metaphor [M ego-moving = 6.027, SD = 0.561; M time-moving = 5.362, SD = 0.742; F_(1, 91)_ = 20.623, p < 0.01]. Conversely, when green advertisements used the loss-framing, the time-moving metaphor was more effective in enhancing participants' green purchase intentions compared to the ego-moving metaphor [M ego-moving = 5.349, SD = 0.885; M time-moving = 5.886, SD = 0.605; F_(1, 87)_ = 12.840, *p* < 0.05]. Therefore, hypotheses H1, H1a, and H1b were successfully validated once again.

##### 3.6.3.5 Mediation effect test

**Figure 4:**
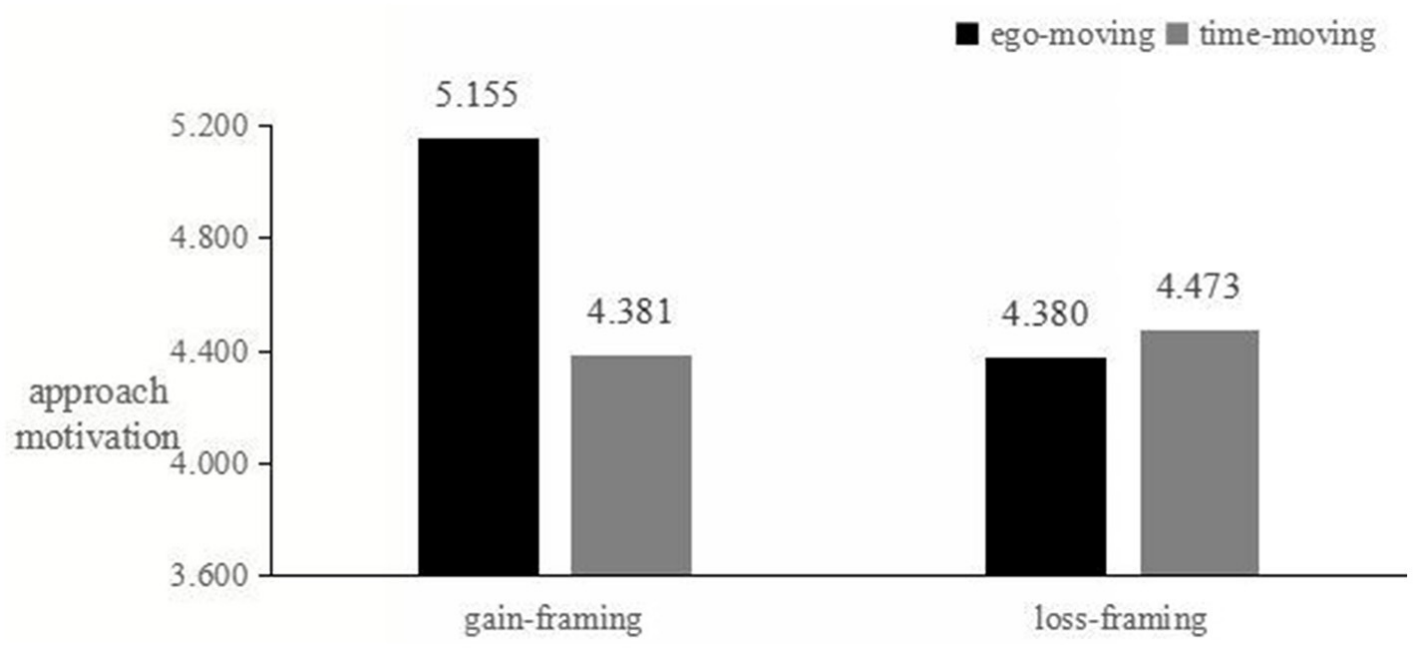
Approach motivation level.

**Figure 5:**
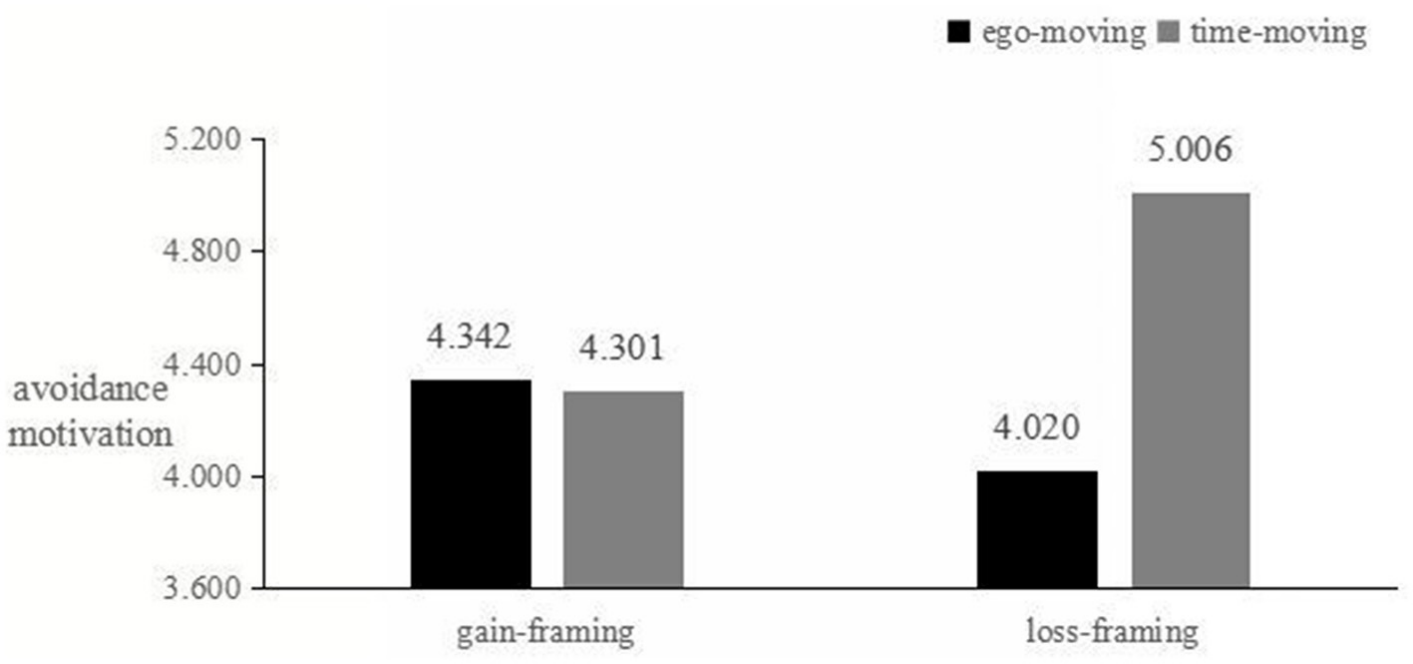
Avoidance motivation level.

The reliability of the approach motivation scale (BAS scale) was a = 0.898, and the reliability of the avoidance motivation scale (BIS scale) was a = 0.902. First, an ANOVA was conducted to examine the matching effect between message framing and time metaphors on approach motivation. The results showed a significant matching effect [F_(1, 179)_ = 15.873, *p* < 0.01]. Specifically, when green advertisements used a gain-framing, the ego-moving metaphor triggered higher approach motivation compared to the time-moving metaphor [M ego-moving = 5.155, SD = 0.677; M time-moving = 4.381, SD = 0.844; t_(91)_ = 4.885, *p* < 0.01]. However, when green advertisements used a loss-framing, there was no significant difference in approach motivation between the ego-moving and time-moving metaphor [M ego-moving = 4.380, SD = 0.748; M time-moving = 4.473, SD = 0.651; t_(87)_ = −0.628, *p* > 0.05]. Next, the matching effect between message framing and time metaphors on avoidance motivation was also significant [F_(1, 179)_ = 12.535, *p* < 0.05]. When green advertisements used a loss-framing, the time-moving metaphor triggered higher avoidance motivation compared to the ego-moving metaphor [M ego-moving = 4.020, SD = 1.295; M time-moving = 5.006, SD = 0.511; t_(87)_ = −4.666, *p* < 0.01]. However, when green advertisements used a gain-framing, there was no significant difference in avoidance motivation between the ego-moving and time-moving metaphor [M ego-moving = 4.342, SD = 1.040; M time-moving = 4.301, SD = 0.924; t_(91)_ = 0.199, *p* > 0.05]. These results confirm that the matching effects of the message framing and time metaphors significantly influence both approach and avoidance motivations, depending on the type of message framing and time metaphor used, as shown in Figures 4, 5.

**Figure 6:**
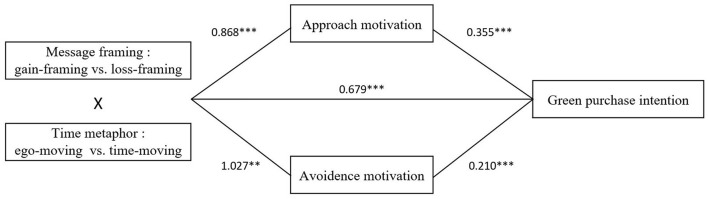
The mediating path of the matching effect between message framing and time metaphor. *p < 0.05, **p < 0.01, and ***p < 0.001.

Utilizing Hayes (2013) PROCESS mediation analysis, Model 8 was employed to perform a Bootstrap test with 5,000 resamples, with green purchase intention designated as the dependent variable. This test investigated the mediating roles of approach and avoidance motivations within a 95% confidence interval. The results revealed that approach motivation mediated the association between message framing and time metaphors with consumers' green purchase intentions (LLCI = 0.438, ULCI = 1.297, excluding 0). Likewise, avoidance motivation also acted as a mediator (LLCI = 0.455, ULCI = 1.599, excluding 0), thereby confirming hypothesis H2. These results are illustrated in Figure 6. Further investigation of approach motivation's mediating effect under varying message framing in green advertisements showed that in the gain-framing group, approach motivation's mediation was significant (LLCI = −0.479, ULCI = −0.114, excluding 0). In contrast, in the loss-framing group, approach motivation did not have a significant mediating effect (LLCI = −0.061, ULCI = 0.158, including 0). This suggests that approach motivation mediates the effect of time metaphors on green purchase intentions when green advertisements using gain-framing. Conversely, when the advertisements using loss-framing, approach motivation does not serve as a mediator, thus supporting hypothesis H2a. When examining the mediating effect of avoidance motivation across different message framing, the results indicated that in the loss-framing group, the mediating role of avoidance motivation was significant (LLCI = 0.081, ULCI = 0.361, excluding 0). However, in the gain-framing group, this mediating effect was not significant (LLCI = −0.104, ULCI = 0.080, including 0). These findings imply that when green advertisements utilize a loss-framing, avoidance motivation mediates the influence of time metaphors on green purchase intentions. Yet, when gain-framing are used, avoidance motivation does not mediate this effect, confirming hypothesis H2b.

## 4 Discussion

With the rapid rise of green consumption, green advertising has become an important medium for companies to communicate product information to consumers (Wang and Li, 2022). Its content and presentation methods can effectively stimulate consumers' attitudes toward green products and their purchase intentions (Teguh and Ignatia, 2022). As a key mode of information presentation in green advertising, the message framing and temporal information play significant roles in profoundly influencing consumers' cognition and behavior (Ni et al., 2022; Sun and Chen, 2024). This study, based on prospect theory and the perspective of temporal movement, designed two experimental scenarios to explore the impact of message framing and time metaphors on consumers' approach-avoidance motivation and green purchase intentions, providing in-depth insights into consumers' green purchasing decisions. The specific findings are as follows.

Previous research has separately validated the effects of message framing and time metaphors on consumer purchasing decisions. However, the results of Experiment 1 indicate a significant matching effect between message framing and time metaphors in green advertising, influencing consumers' intentions to purchase green products. Specifically, when the green advertisement employs a gain-framing, the use of a ego-moving metaphor effectively enhances consumers' green purchase intentions; conversely, under a loss-framing, the employment of a time-moving metaphor significantly boosts purchase intentions. These findings support hypotheses H1, H1a, and H1b. This discovery suggests that marketers can combine gain-framing with ego-movingt metaphor while aligning loss-framing with time-moving metaphor to more effectively convey information about green products. The coordination of message framing and time metaphor can enhance consumers' willingness to purchase green products, thereby increasing sales of green products for companies.

Most of the previous research has focused primarily on examining the mediating roles of attitudes, beliefs, perceived behavioral control, subjective norms, and personal norms in consumer behavior using the Theory of Planned Behavior (TPB), Consumption Value Theory, and Value-Belief-Norm Theory (Karpudewan, 2019; Confente et al., 2020; Rausch and Kopplin, 2021). In contrast, this study approaches from the perspective of consumer psychological motivation, focusing on the mediating roles of approach and avoidance motivations in the matching effects of message framing and time metaphor. Specifically, when green advertisements use a gain-framing, approach motivation mediates the effect of time metaphor on consumers' green purchase intentions; however, under loss-framing conditions, approach motivation does not mediate the effect of time metaphor on green purchase intentions. Conversely, when green advertisements adopt a loss frame, avoidance motivation mediates the effect of time metaphor on green purchase intentions, while avoidance motivation does not play a mediating role under gain-framing conditions. Therefore, marketers should pay attention to eliciting the corresponding approach and avoidance motivations of consumers when designing green advertisements. Specific psychological motivations can effectively assist consumers in selecting green products and enhance their willingness and behavior to engage in green consumption.

## 5 Conclusion

The information content and presentation methods of green advertising will have a certain impact on consumers' willingness to consume green products. This study integrates prospect theory, the time-motion perspective, the S-O-R (Stimulus-Organism-Response) model and regulatory focus theory to investigate how green advertising message framing (gain vs. loss) and time metaphor (ego-moving vs. time-moving) influence consumers' green purchase intentions. It also examines the mediating role of approach and avoidance motivations. Through two scenario-based experiments, the following key findings were obtained:

First, there is a significant matching effect between advertising message framing and time metaphors on consumers' green purchase intentions. Green advertisements that employ gain-framing to emphasize positive outcomes, paired with ego-moving metaphor, are more effective in stimulating positive purchase intentions. Conversely, advertisements utilizing loss-framing to highlight negative consequences, combined with time-moving metaphor, also elicit stronger green purchase intentions.

Second, approach and avoidance motivations mediate the interaction between message framing and time metaphors. Specifically, gain-framing paired with ego-moving metaphor activates approach motivation by emphasizing positive outcomes, thereby enhancing green purchase intentions. In contrast, loss-framing paired with time-moving metaphor generates avoidance motivation by stressing negative consequences, which also enhances green purchase intentions.

Therefore, the results of this study further elucidate the optimal information matching combination for green advertising, calling on marketers to change consumers' preferences and behaviors by improving green marketing design strategies. The results have a significant impact on green marketing for enterprises. The matching combination of message framing and time metaphors stimulates consumers to generate different psychological motivations, which in turn affects their green decisions. This study not only contributes to enriching the theory, but also provides new directions for explaining consumer decision-making behavior: approach motivation and avoidance motivation. The research results lay the foundation for further research on green advertising marketing, while exploring the boundaries of expanding sustainable consumption research.

## 6 Implications of the study

### 6.1 Theoretical implications

This study enhances the understanding of message framing and time metaphors in green consumption. Previous research largely focused on psychological factors, investigating how various information frames affect consumers' willingness to make green purchases while often neglecting the influence of other advertising elements, such as time metaphors. Unlike earlier studies that concentrated on the selection differences of time metaphors, this research emphasizes their impact on consumer psychology and behavior. By exploring the relationship between time metaphors and information framing, we identify a matching effect that reinforces the theoretical framework and enriches the comprehension of how time metaphors facilitate sustainable consumer behavior, thereby providing a novel perspective on the effectiveness of green advertising.

Additionally, using prospect theory and the concept of time movement, this study evaluates effective combinations of advertising information aimed at enhancing green purchasing intentions in specific contexts. We examine the differential effects of pairing gain-framing with ego-moving metaphor and loss-framing with time-moving metaphor on consumers' green purchase intentions. This analysis offers a comprehensive theoretical perspective on the complexities of consumer processing of green advertising messages, enabling researchers and practitioners to formulate more effective strategies for promoting sustainable consumption.

Finally, this research clarifies why individuals are inclined to respond positively to consistent information. Grounded in the S-O-R model, examines how message framing and time metaphors influence green purchase intentions. The findings not only reinforce the application of the S-O-R theory in studying consumer sustainable behaviors, thereby expanding its research boundaries, but also further validate the theory's applicability across various consumption contexts, highlighting its potential impact in the area of green consumption. Previous studies have primarily focused on perceived green value and processing fluency to explain the influence of external advertising messages on sustainable consumer behavior, often overlooking psychological motivations. Consistent information exposure tends to evoke positive consumer responses, driven by the need for cognitive consistency. This research highlights the mediating roles of approach and avoidance motivations in the matching effects between message framing and time metaphors, deepening our understanding of how green advertising affects consumer decision-making and establishing a foundation for future studies on green consumption intentions.

### 6.2 Practical implications

First, this research can assist companies in formulating persuasive green advertising strategies, thereby boosting the market share of green products. When designing green advertisements, marketers should pay attention to matching message framing with time metaphors. Specifically, when a business aims to convey the benefits of a green product to consumers, it should use ego-moving metaphors to increase the urgency of anticipating benefits (e.g., “enjoy steep discounts” or “gradually achieve green environmental goals”). Conversely, when conveying the harms of traditional products, businesses should use time-moving metaphors to enhance the urgency of impending loss (e.g., “environmental issues are imminent” or “limited-time discounts are about to expire”).

Second, companies should also focus on stimulating consumers' psychological motivations when designing green advertisements, which can help increase the purchase rate of green products. Marketers can use different background elements depending on the ad-message combination to highlight the key attributes of green products and trigger corresponding approach or avoidance motivations, thus effectively enhancing green purchase intentions.

Third, this approach contributes to raising consumers' environmental awareness, fostering green consumption habits, and encouraging low-carbon lifestyles. Companies can include content in their green advertisements or online marketing posts that explain the consequences of environmental pollution or the necessity of protecting the ecosystem (e.g., “the harmful effects of excessive plastic pollution”). By improving consumers' environmental knowledge, businesses can increase consumers' sense of responsibility and urgency toward environmental protection, thereby effectively encouraging them to engage in green consumption behavior. Limitations and future research directions.

## 7 Limitations and future research directions

This study investigates the internal mechanisms by which green advertising message framing and time metaphors influence consumers' green purchase intentions but acknowledges some limitations. First, while most businesses use short video ads on new media platforms for promotion, this study used a combination of text and images for easier manipulation, which may not fully reflect real marketing scenarios. Future research could use video ads to examine potential differences in the impact of green advertising strategies on consumers' green consumption decisions.

Second, the study employed a questionnaire survey, creating purchase scenarios and collecting data from participants. However, the green purchase intention reported by participants may differ from their actual behavior in the real world. Future studies could use real green consumption data from online shopping platforms to more comprehensively explore the relationship between green advertising strategies and consumer behavior.

Lastly, this study did not examine potential moderating factors in the interaction between green advertising message framing and time metaphors. Other factors may influence this relationship. Future research could incorporate additional moderating variables (e.g., construal level, environmental concern, moral emotions) to explore whether these matching effects vary under different conditions.

## Data Availability

The original contributions presented in the study are included in the article/supplementary material, further inquiries can be directed to the corresponding author.
